# Supplementary material for: Effects of short-term arm immobilization on motor skill acquisition
Source: PLoS One. 2022 Oct 14;17(10):e0276060. doi: 10.1371/journal.pone.0276060 (PMC9565666; doi:10.1371/journal.pone.0276060)
Supplement: Attachment — Submitted filename: ResponseToReviews.docx [file pone.0276060.s002.docx]

Response to Reviewer Comments:

1) To test the homogeneity of variance, the authors should use Levene’s test, not Q-Q plots

**Thank you for this suggestion. We performed Levene’s test to assess homogeneity for each outcome measure across timepoints for both left and right hand data. The variances were not significantly different for any of the outcome measures. This information has been added to the manuscript.**

2) When reporting the degree of freedom, they should report both the numerator and the denominator

**Thank you for pointing this issue out. The numerator and denominator of the degrees of freedom (DFn, DFd) have been added to the tables.**

3) The references have to be homogenized (e.g., l.68: A study by Opie, Evans (22)…)

**The references have been edited throughout to improve consistency in formatting.**
